# Spontaneous coronary artery dissection and fibromuscular dysplasia: insights into recent developments

**DOI:** 10.3389/fcvm.2024.1409278

**Published:** 2024-05-31

**Authors:** Ayah Eltabbakh, Ahmed Khudair, Aiman Khudair, Salim Fredericks

**Affiliations:** Department of Medicine, Royal College of Surgeons in Ireland—Bahrain, Busaiteen, Bahrain

**Keywords:** spontaneous coronary artery dissection, fibromuscular dysplasia, acute coronary syndrome, SCAD, PHACTR1

## Abstract

Spontaneous coronary artery dissection (SCAD), an uncommon cause of acute coronary syndrome, continues to be a poorly understood disease predominantly affecting females. It is characterized by an abrupt separation in the coronary arterial wall due to intramural bleeding. Fibromuscular dysplasia (FMD) is a non-atherosclerotic arteriopathy manifesting in medium and small-sized arteries. It is a concomitant disease found among SCAD patients. In some studies, FMD prevalence in SCAD patients ranges between 25%–86%, which can be explained through varying screening techniques or modalities. The potential association has been elucidated in some studies; notably, not only has a genetic link been recently delineated between SCAD and FMD, but there is data to suggest that FMD not only can predispose to SCAD but can also be a potential predictor of its recurrence. However, a clear-cut correlation between the two has still not been established due to conflicting reports in the literature. To further dive into its pathology, it is crucial to highlight the importance of systematic screening in SCAD in order to identify associated risk factors and to be used as a method of FMD detection in such patients. Together, the two pathologies pose unique challenges in understanding its pathophysiology, diagnosis and management, as there is no clear evidence of a definitive treatment plan for patients with SCAD and FMD. A potentially beneficial modality of management is physical exercise, which is currently understudied in the long-term approach to treatment for patients with concomitant SCAD and FMD. Limited research in this field brings disadvantages to the understanding of the association between these two diseases, in order to give rise to better management recommendations. This mini-review aims to highlight the recent developments in the association between SCAD and FMD, its potential genetic association and some insights in screening, diagnosis, and management.

## Search strategy

For this review, a literature search was conducted between February 2024 and March 2024 utilizing Google Scholar and PubMed. The search included keywords, not limited to “Spontaneous coronary artery dissection”, “SCAD”, “Fibromuscular Dysplasia”, “FMD”, “Spontaneous coronary artery dissection and Fibromuscular Dysplasia”, and “PHACTR1”. Articles were included based on relevancy, with a higher emphasis on more recent publications within the past five years.

### What is SCAD

#### Definition

Acute coronary syndrome (ACS) remains to be the leading cause of mortality worldwide (1). It is described as an acute decrease in blood flow to the heart that encompasses a variety of conditions including unstable angina, ST-segment elevation myocardial infarction (STEMI), and non-STEMI (2).

An increasingly important cause of ACS is spontaneous coronary artery dissection (SCAD) which is defined as the spontaneous rupture of a coronary artery wall, not caused by trauma, iatrogenesis, or atherosclerosis, resulting in the formation of a false lumen and intramural hematoma (3–5).

#### Epidemiology

First described in 1931, SCAD is approximated to cause 1%–4% of ACS (6–11). The majority of SCAD cases affect females (87%–95%), primarily under the age of 50 and classically without typical cardiovascular risk factors (11–14). Significantly, in females <60 years old, SCAD comprises 35% of ACS (7, 15, 16). It is also regarded as the predominant cause of MI within pregnant individuals (43%) (13). SCAD has historically been considered rare, partly explained due to its underdiagnosis and low clinical suspicion of myocardial infarction (MI) within the presenting population. However, recently, the rarity of SCAD has come into question (11, 17). One contributing factor to this rise in incidence could be due to the improved knowledge of its appearance on angiography and enhanced physician awareness as opposed to a true elevation of SCAD within the general population (17).

#### Pathogenesis

There are two prevailing hypotheses detailing the pathogenesis behind SCAD. These are named the “inside-out” and “outside-in” hypotheses, the former is described as subintimal blood extravasation following a disruption in the intima of the vessel wall that leads to the generation of a false lumen within the tunica media (5, 14, 18). The latter hypothesis describes hemorrhage and subsequent intramural hematoma formation within the medial wall of a vessel due to a bleed from the vasa vasorum devoid of an intimal tear (5, 14, 18). Ultimately, compression of the lumen, regardless of the mechanism, leads to ischemia which is the cause of MI in SCAD patients (14, 19).

#### Types of SCAD

SCAD is split up into 4 types. On angiography, type 1, regarded as pathognomonic of this disease, is recognized as a flap of the intima that allows contrast dye to enter the false lumen of the vessel wall (20–22). Type 2 and 3 appear as a narrowed coronary artery wall, however, they differ from type 1 due to the absence of an intimal flap hence the lack of staining of the vessel wall (20). Type 2, typically a >20 mm artery narrowing, can be further subdivided into two types, type 2A and type 2B (21, 22). The former is characterized by a stenosed coronary artery with the presence of normal segments seen proximally and distally to the lesion while the latter is characterized by a stenotic segment continuing to the distant end of the artery (20, 22, 23). Type 3 can be misconstrued as atherosclerosis, as it appears as a focal stenosis (4, 21). This type is a challenging diagnosis that warrants the usage of intracoronary imaging such as optical coherence tomography (OCT) and intravascular ultrasound (IVUS) (4, 21). Type 4 was recently introduced and describes a complete, usually distal, occlusion of a coronary vessel when embolic etiologies have been ruled out (22, 24).

#### Diagnosis

The gold standard for diagnosing SCAD is the usage of coronary angiography (25). Aided by the recent introduction of the Saw angiographic classification, this has refined the accuracy and identification of SCAD diagnosis (5, 26, 27). However, invasive techniques in an already vulnerable vessel can lead to further damage and must be utilized with caution (19). Furthermore, if coronary angiography does not yield a definitive diagnosis, OCT or IVUS may be used. Another less commonly utilized approach due to its insufficient sensitivity and false-negative rate is cardiac computed tomography angiography, but it has been used as a non-invasive modality for SCAD follow-up (25, 27).

#### Management

Restoration of sufficient perfusion distal to the SCAD lesion is the mainstay of acute SCAD management (28). SCAD management has differed over the years. However, recently, the American College of Cardiology (ACC) and the American Heart Association (AHA) have recommended conservative approaches for stable SCAD patients (28, 29).

A retrospective cohort study published by Feldbaum et al., demonstrated that there has been a significant rise in the usage of a conservative approach before 2013–2019 going from 35% to 89% (30). A recent systematic review demonstrated that the conservative approach has been deemed as the favored treatment modality (3). The reasons opposing invasive strategies are due to the fact that vessels that undergo dissection in SCAD spontaneously heal within 4–6 weeks and that revascularization procedures carry a significant rate of failure along with decreased outcomes (25, 28). While invasive approaches are usually reserved for unstable patients such as those with arrhythmia, hemodynamic instability, vessel occlusion, and ongoing cardiac ischemia (25, 28). A limitation in the literature surrounding SCAD management is the lack of randomized controlled trials, therefore this is paramount to guide the future of SCAD management (28, 31).

#### Risk factors

**Figure 1:**
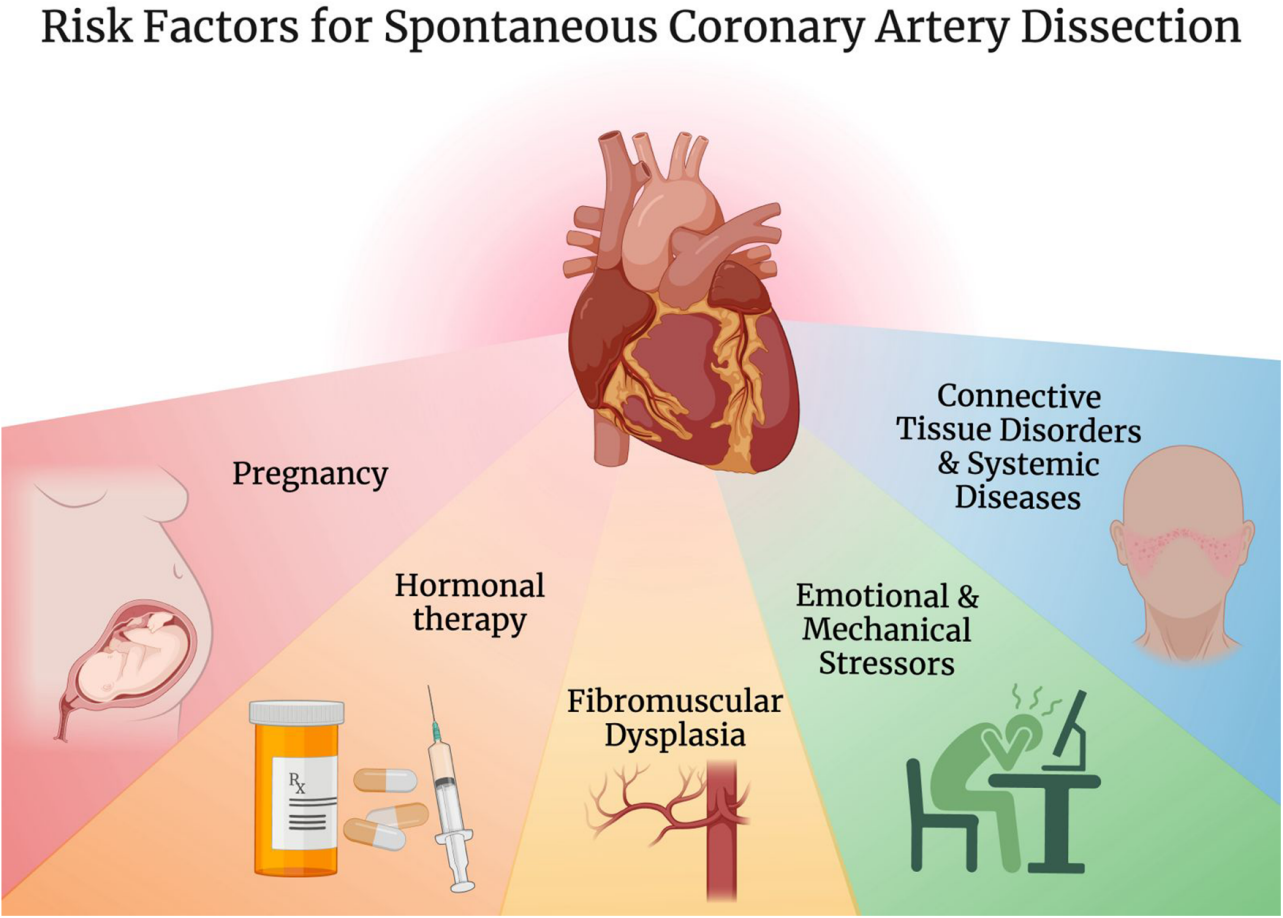
Risk factors for spontaneous coronary artery dissection. Created with BioRender.com.

A variety of predisposing conditions have been described for SCAD including pregnancy, hormonal therapy, connective tissue disorders such as Marfan syndrome and Ehler-Danlos syndrome, systemic diseases such as systemic lupus erythematosus as well as vasculitides like Takayasu arteritis and granulomatosis with polyangiitis, fibromuscular dysplasia (FMD), significant emotional and mechanical stressors (Figure 1) (3, 6, 32–35). FMD is of particular interest, initially described in association with SCAD in 2011, and is now seen concomitantly in more than 50% of SCAD patients in large cohorts (6, 15, 35–38).

#### Pregnancy

As mentioned previously, SCAD is a predominant cause of MIs in pregnancy, occurring in up to 43% of pregnant individuals (5, 13). Of all SCAD cases, approximately 10% are associated with pregnancy, occurring in 1.81 out of 100,000 pregnancies, with the post-partum period implicated in 72.5% in pregnancy-associated SCAD (P-SCAD) (5, 13, 39–41). In fact, pregnancy is a precipitating factor for SCAD with several theories tying pregnancy to SCADs' pathogenesis (5).

In pregnancy, the excess progesterone may lead to decreased collagen synthesis, medial collagen wall degradation, and loss of elastic fiber corrugation, causing weakening of the tunica media, hence predisposing to an arterial dissection (13, 42, 43). Similarly, an increase in estrogen may cause medial wall breakdown and weakening of the vaso-vasorum via the induction of matrix-metalloproteinases (MMP) release, predisposing to an artery prone to dissection (1, 7, 8). Additionally, increased blood volume, heart rate, and cardiac output within pregnancy promote shear forces on the arterial wall (13, 43). These hormonal and hemodynamic changes may persist for up to 6 months after delivery, keeping risk of SCAD relevant for those postpartum (5, 13, 44).

Management of P-SCAD involves a multidisciplinary approach considering the patient's hemodynamic stability, the location and magnitude of myocardium impacted, and fetal well-being (43). While conservative management is of choice in stable patients, this modality is sophisticated with some drugs being unsafe in different pregnancy stages (40, 42, 43). Thrombolysis in pregnancy is a relative contraindication, and its use is generally avoided in pregnancy due to the risk of propagating the dissection, however, this option remains controversial (42, 43). Generally, in those with ongoing ischemia in a single vessel, percutaneous coronary intervention (PCI) may be chosen (42, 43). However in those with multi-vessel involvement, left main stem dissection, or those who have failed PCI or medical therapy, coronary artery bypass grafting may be selected (42, 43).

### Fibromuscular dysplasia

#### Definition

FMD, first described in 1938, is an uncommon, non-atherosclerotic, non-inflammatory, segmental, and idiopathic arteriopathy that affects muscular small and medium-sized arteries that result in stenosis (45–50).

#### Epidemiology

FMD most commonly occurs in females (82%–95%) usually presenting in middle age with a mean age of diagnosis at 43–53; however, it can occur at any age (51, 52).

#### Pathology

FMD primarily affects the renal vasculature, comprising around 75% of these patients but it also can affect the carotids, iliacs, and vertebral arteries, however, it has been described to affect virtually every artery (48, 50, 53, 54). FMD can present in a variety of ways, from asymptomatic hypertension, all the way to dissections, occlusions, stenosis, and aneurysms (50, 54, 55).

Previously, the classification of FMD was primarily histological, manifesting as intimal, medial, and adventitial fibroplasia. However, there has been a shift towards angiographic classification (55, 56). The two subtypes, focal and multifocal, with multifocal being the most common comprising over 80% of FMD cases (51). On angiography, it displays the classical “string-of-beads” appearance due to the alternation of stenotic and dilated segments (51, 57). While focal is a constriction occurring in one particular area of the vessel (55).

#### Diagnosis

The prevailing symptoms upon presentation for FMD include hypertension, dizziness, tinnitus, and headache. However, depending on the underlying pathology of FMD, such as dissection or aneurysm, symptoms such as abdominal pain or chest pain may prevail (52, 58) Furthermore, the location of FMD will also determine what symptoms appear. Previously, FMD diagnosis was performed through histological findings, however, after the publication of the international consensus statement by the European and AHA, imaging findings are now central to FMD diagnosis. The gold standard for diagnosis of FMD is the usage of catheter-based digital subtraction angiography (55). However, the emergence of non-invasive modalities such as CT angiography, magnetic resonance angiography, and duplex ultrasonography are now taking over the role of angiography (52).

#### Treatment

FMD continues to have no definitive cure, however, many management modalities can improve patient outcomes (59). Treatment is typically aimed at the vascular bed involved (59). Vital players in management are the control of blood pressure as well as the prevention of thrombosis or thromboembolic events (56). Furthermore, other common symptoms in FMD such as pulsatile tinnitus and headache must also be addressed (57). However, for those with complications such as aneurysms or dissections, invasive techniques may be incorporated in their treatment regimen (59).

#### Association between SCAD and FMD

As defined earlier, SCAD and FMD are both diseases that affect specific blood vessels in the body. While they primarily affect different types of arteries, evidence suggests a strong association between the two conditions due to their similar pathophysiology (60) Recent studies mention that up to 86% of SCAD patients have FMD in a non-coronary artery (29). However, almost 25% of SCAD patients present with no clinical evidence of FMD. As such, this prompts the question on the connection between these two diseases and whether they are separate but have related arteriopathy or perhaps a milder form of FMD exists that has yet to be detected through diagnosis (5). While these diseases can coexist in some patients, the relationship is not fully understood. Therefore, with more investigations and an increase in the number of individuals with both SCAD and FMD, it has been found that sex steroids and genetics play a role in the development of these diseases.

#### Genetics

During the last few years, an understanding into the genetic relation between SCAD and FMD has been discovered to further explain their connection (37). As of today, a single genetic locus has been identified to link both conditions (37). Due to its pleiotropic nature, the phosphatase and actin regulator 1 (PHACTR1) is known to increase the risk of several vascular and coronary artery diseases, including FMD and SCAD (61–63). This gene encodes a protein that adheres to actin and protein phosphatase 1 which contributes to endothelial tube formation as well as endothelial survival (64). Additionally, it promotes the calcification of blood vessels in vascular smooth muscle cells and is crucial in angiogenesis (64). Therefore, the PHACTR1 gene has been identified to be the cause of several cardiovascular diseases. Based on Kiando et al.'s studies, PHACTR1 has been identified on endothelial and smooth muscle cells in FMD patients (65). As such, it is suggested that the above mentioned gene might support the idea that both SCAD and FMD could stem from the same underlying disease (66).

**Figure 2:**
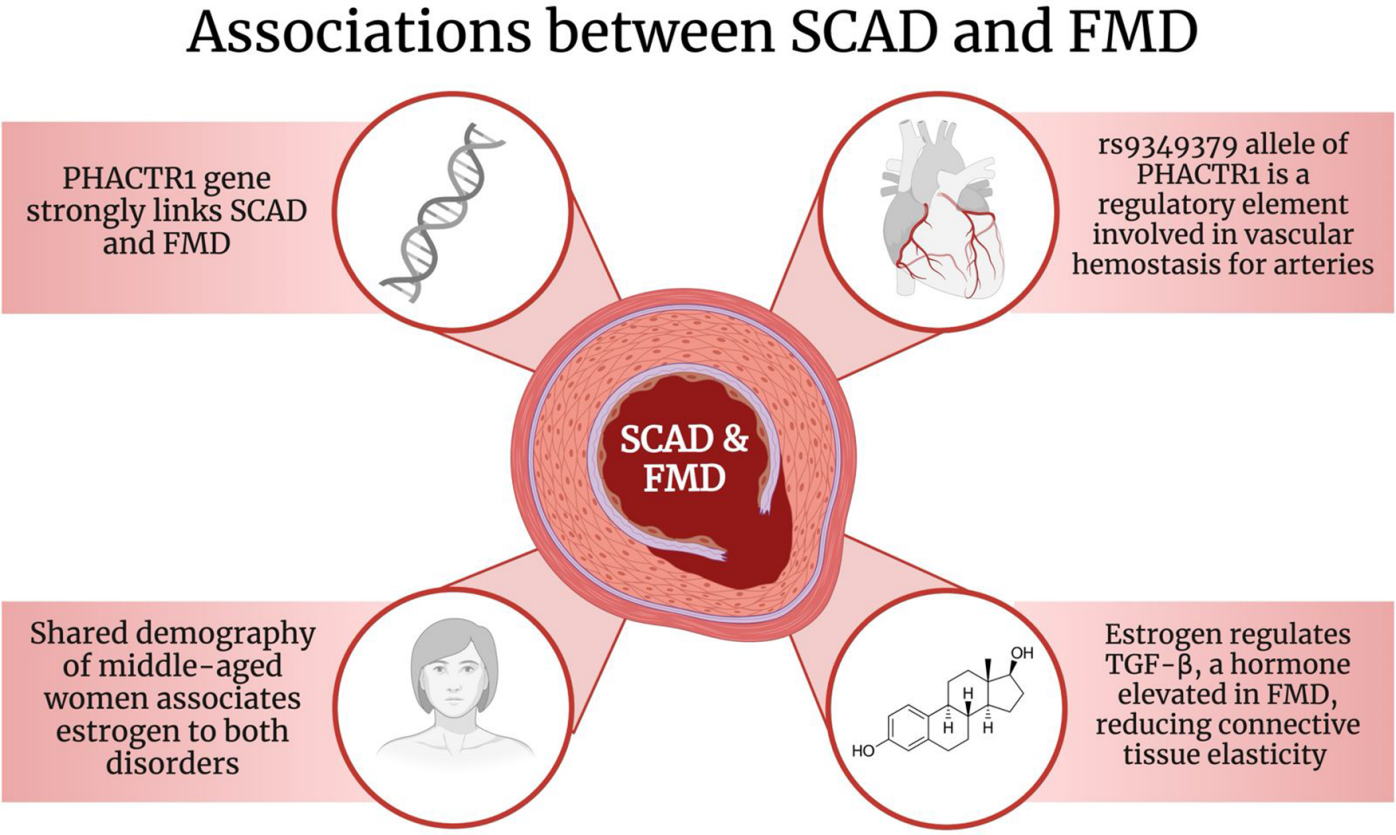
The associations between SCAD and FMD. Created with BioRender.com. SCAD: Spontaneous Coronary Artery Dissection; FMD: Fibromuscular Dysplasia; PHACTR1: phosphatase and actin regulator 1; TGF-*β*: Transforming growth factor-beta.

In particular, the allele rs9349379 on the PHACTR1 gene has been found to be associated with both SCAD and FMD (Figure 2). A study conducted by Georges et al. discovered that the rs9349379 allele was found in 83 FMD patients (67). As part of its functions, the rs9349379 allele is a specific regulatory element for arteries (67). Its genetic makeup plays a role in binding transcription factors, myocyte-specific enhancer factor 2 (MEF2), making it an important group of regulators for vascular homeostasis (67, 68). Furthermore, Gupta et al. suggested that the rs9349379 allele enhances the expression of endothelial-1 protein (ET-1), a potent endogenous vasoconstrictor, and a cell proliferation regulator (6, 61, 69, 70). Nonetheless, this protein has been discovered to have multiple effects on vessels, particularly on arterial tone and remodeling (37). Due to its effects on vasculature, it is possible that it may play a role in the pathophysiology of SCAD as well as increase its risk (6, 66).

The variant A of the rs9349379 allele specifically has been recently associated with FMD and SCAD (37, 71, 72). However, the variant rs9349379-G has been identified to be reduced in coronary arteries and is connected to atherosclerosis (71, 72). A study reported by Adlam et al. suggested that the risk of SCAD associated with the rs9349379-A allele was explained by a lower expression of endothelin-1 (ET-1) (61, 66). In fact, healthy patients had the G variant of the allele and had a higher expression of ET-1 whereas SCAD individuals had the variant A and a lower expression of the protein (72). As ET-1 is associated with vasoconstriction and remodeling, it is suggested that reduced levels would be beneficial, however the connection is unclear and further investigations are needed (71). Despite that, evidence strongly suggests and explains the involvement of the PHACTR1 gene and particularly the rs9349379 allele in the association between SCAD and FMD as well as its function on different arteries.

#### Sex-steroids and transforming growth factor-beta

Other than genetics, both conditions have been found to be sex biased. Although rare, SCAD is 9 times more likely to occur in women, representing more than 90% of its cases (29, 37, 67, 73). Additionally, like SCAD, FMD—specifically multifocal FMD—predominantly affects women (37). According to Rana et al., 12 out of 100,000 FMD in the United States patients are females while males only represent 0.004% of that population (74). As females have a higher prevalence in both diseases, it is suggested that being exposed to endogenous or exogenous estrogens may increase the likelihood of developing either condition (75). In fact, it has been discovered that estrogen has an impact on cardiovascular diseases and has specific effects in regulating vascular reactivity as well as blood pressure (73).

Estrogen is a potent vasodilator (26). Estrogen's action is triggered by the release of nitric oxide (NO), a potent vasodilator (26, 76). As such, NO production leads to the inhibition of the vasoconstricting peptide, ET-1 (76). In addition, estrogen plays a fundamental role in the formation of blood vessels (73). This is due to the fact that it regulates biochemical mediators involved in angiogenesis such as MMPs and transforming growth factor beta (TGF-*β*) (73, 77). As previously mentioned, connective tissue disorders such as Marfan and Ehlers-Danlos syndromes have been found to be risk factors for both SCAD and FMD (78, 79). According to a study done by Maas et al., TGF-β appeared to be elevated in Marfan syndrome (29, 73). Additionally, several sources confirm the presence of elevated TGF-β in many SCAD and FMD patients and therefore might suggest the hormone's involvement in the conditions (14, 37, 57, 62, 74, 80).

To further explain its connection, TGF-β causes an increased collagen production; thus it will reduce connective tissue elasticity (72, 73). As arterial tortuosity is a common manifestation of both SCAD and FMD, many sources suggest that TGF-β activity has an effect on it, which may lead to arterial weakening (5, 73). Moreover, estrogen mediates the release of MMPs, an enzyme important in the breakdown of extracellular matrix (ECM) and the degradation of arterial walls, affecting its structural integrity (6, 72, 73, 81). As such, MMPs have been found to be crucial in new blood vessel formation and ECM remodeling. Therefore, this might explain its involvement in the pathogenesis of SCAD by weakening arteries and causing them to rupture spontaneously (72).

#### Screening

Both SCAD and FMD share the same demographic of patients, with the majority being young to middle aged females (37). Upon diagnosis, a full screening was done on SCAD patients and results showed that 63% of these individuals had concomitant FMD (37, 82). In fact, Hayes et al.'s study suggests that SCAD is an early manifestation of systemic arteriopathy (5). While other studies imply that SCAD is associated with extra coronary abnormalities such as FMD and therefore, it is suggested that FMD predisposes SCAD (83). Given the association between the two conditions, genetic screening for FMD in SCAD patients may be beneficial for risk assessment as to guide management and prevent late manifestations of extra coronary diseases. It is important that this be performed through a head-to-pelvis CT angiography or magnetic resonance angiography (72, 75, 82).

## Conclusion

As awareness of SCAD and FMD has been increasing over the years, it is important to look into their association, as well as the current management and risk factors. SCAD is an important cause of ACS, described by a sudden rupture of a coronary artery which leads to a false lumen and intramural hematoma. In fact, SCAD represents 1%–4% of ACS cases, with a higher prevalence in middle aged women. Additionally, SCAD is usually mistaken for atherosclerosis, making it difficult to diagnose and as such it is classified into four types depending on angiographic findings. Furthermore, the gold standard imaging for SCAD is coronary angiography and in particular non invasive methods such as CT angiography. Conservative management is typically the best approach as SCAD lesions heal spontaneously within weeks. Moreover, common risk factors for the disease include pregnancy, hormonal therapy and connective tissue disorders such as FMD. It commonly affects renal arteries and usually presents with hypertension. Just like SCAD, FMD can be detected through non-invasive techniques like CT angiography while predominantly affecting women. As such, management involves symptoms and blood pressure control. As both conditions have similar pathophysiologies, a strong genetic link has been found between SCAD and FMD, particularly involving the PHACTR1 gene. In addition, mechanisms including estrogen and TGF-β have also been found to be involved in both conditions. Finally, screening for FMD is important in SCAD patients as it may help with risk assessment and refine different management plans.
